# Our Book Table

**Published:** 1876

**Authors:** 


### OUR BOOK TABLE.

Cholera epidemic in the United States. The introduction of epidemic cholera through the agency of the merantile marine; suggestions of measures of prevention. By John M. Woodworth, M.D., Supervising Surgeon United States (Merchant) Marine Hospital service.

Reports prepared under the direction of the Surgeon-General of the army.

A.—History of the cholera epidemic of 1873 in the United States. By Ely McClellan, M.D., Assistant Surgeon United States Army.

B.—History of the travels of Asiatic cholera. By John C. Peters, M.D., of New York City, and Ely McClellan, M.D., Assistant Surgeon United States Army.

C.—Bibliography of Cholera. By John S. Billings, M.D., Assistant Surgeon United States Army, Washington. Government Printing Office, 1875.

We have carefully given the title pages of this work, in order more clearly show the reader its object and scope. It comprises a volume of over one thousand pages, and embraces facts, statistics, etc., of the greatest value. It is, beyond doubt, the fullest and most thorough compilation on epidemic cholera extant. It is exceeding creditable to the medical staff of the army and to the Government.

Hospital Plans. Five essays relating to the construction, organization and management of hospitals, contributed by their author for the use of the John's Hopkin's Hospital, of Baltimore. New York, William Hood & Co., 27 Great Jones street, 1875.

This volume, as seen above, consists of five essays, contributed by John S. Billings, M.D., Norton Folsom, M.D., Joseph Tones, M.D., Caspar Morris, M.D., and Stephen Smith, M.D., and an appendix by John R. Niennsee, F. A. I. A. It is copiously illustrated. To our mind, it covers hospital construction and management completely, and is an invaluable work to the physician and citizen, in view of the construction and management of hospitals in this country.

Archives of Dermatology. Vol. I. We are indebted to G. P. Putnam's Sons, New York, for this beautifully bound copy of the first volume of Archives of Dermatology. This is, as our readers know, a quarterly journal of skin and venereal diseases, edited by L. Duncan Balkney, A.M., M.D., with a long list of distinguished colaborators.

The *Archives* is a most valuable journal, and will be of great service to professional brethren in the diagnosis and treatment of skin and venereal diseases.

Transactions of the twenty-fifth anniversary meeting of the Illinois State Medical Society, held in the city of Jacksonville, May 18th, 19th and 20th, 1875.

Our brethren of Illinois show their "faith by their works." This State Society is in a most flourishing condition. The papers are numerous, and show their authors to be fully abreast with the medical service of the times. The report on theory and practice, by P. C. Hamill, M.D. of Chicago, abounds with practical ideas and sound treatment. He shows the value of phytolaica decandra, and guaiaci resini in the treatment of subacute and chronic forms of rheumatism and in trissillibis. He has had "beautiful" results in the treatment of these diseases by the remedies named, which we would gladly, if we had space, give room for in our pages. To a patient whose "knees, ankles and wrists were swollen and painful—unable to straighten himself on account of pain in his back"—he gave "tinct. phytolacca and tinct. graiacum, equal parts; one drachm three times a day (*i.e.*, milk), and, between the doses, a dessert spoonful of the following solution: Acet. potass., six drachms; mint water, four drachms, with half a drachm of bicarb. soda, in a small glass of water, and to take 10 grains of blue mass at bed time. He took up the solution, and four ounces of the mixture, and was relieved." To a patient aged 6 years, with acute tonsillitis, he ordered the following mixture: "One teaspoonful to be given every two hours; chlorate potass., drachms, one-half; tr. guaiacum, drachms, two: tr. phytolacca, drachms, one; mucil. acacia, q. s.; honey, q. s.; cinnamon water, q. s.; to make ounces three. And if there should be an occasion of fever, one drop of tinct. aconite root, to be given half way between the doses.

The volume is handsomely bound, for which we are indebted to the Secretary, Dr. T. Davis Fitch, Chicago.

Transactions of the Medical Society of the State of West Virginia. These transactions show a number of excellent papers. The Society is composed of earnest men, reflecting credit upon the profession in the State. Dr. William M. Dent, of New-bury, furnished us with our copy.

Physicians' Combined Call Book and Tablet. This is one of the best and most convenient visiting lists we have ever seen. It embraces the doses of medicines, abbreviations, etc., for reference. Those wishing it should address Dr. Ralph Walsh, 324 Four and a Half Street, Washington, D.C.

Vick's Floral Guide is, as usual, the handsomest that has been issued. It contains cuts, flowers, scenery, etc., as beautiful as heart could wish. Vick's seed, both floral and vegetable, cannot be excelled. Send for the catalogue and Floral Guide, before ordering seeds elsewhere. 25 cents is the price. James Vick, Rochester, N. Y.

*Books Received.*—We are indebted to H. C. Lee, of Philadelphia, for Physiology, by J. C. Dalton, M.D. (sixth edition.) Taylor on Poisons, and Lee's Lectures on Syphilis, too late for notice in this issue.

Transactions of the New Hampshire Medical Society. Held at Concord. A number of papers appear in these transaetions. The reports on Gynæcology; on Fractures and Plastic Splints; and Sanitary Measures in Rural Districts, are worthy of a wider circulation than can be given them in these transactions. Our thanks are due Dr. G. P. Conn, of Concord, for the volume.

*Pamphlets.*—A series of American Clinical Lectures. Edited by E. C. Leguin, M.D.

Vol. I., No. 1. On diseases of the hip joint: by Lewis A. Sayre, M.D. Vol. I., No. ix. Peritoritis: By Alfred L. Loomis. Published by G. P. Pulman's Sons, New York.

Morrison Colorado, United States Army. Its mineral waters and climate. By S. Edwin Salley. McKittrick & Co., St. Louis.

Annual Oration before the Medical and Chirurgical Faculty of Maryland, by Joseph M. Toner, M.D.

Treatment and removal of febroids from the uterus, by traction. By Thos. Addes Emmet, M.D., New York.

A retrospect of the struggles and triumphs of Ovariotomy in Philadelphia, with some additional remarks on allied subjects. By Washington L. Atlie, M.D., Philadelphia.

Sceleritis Siphilitica: Its pathology, course and treatment. By Fred. R. Sturgis, M.D., New York. G. P. Pulnam's Sons.

Cases of Hysteria. Nervous themia, spinal irritation and allied affections; with remarks by Geo. M. Beards, New York.

Migrants and sailors considered in their relation to public health. By John M. Woodworth, M.D., and Heber Smith, M.D.

Review of Prof. Palmer's statements respecting the relations of himself and colleegues to Homœpathy in the University.

Hermaphrodism, from a medico-legal point of view. Chicago, W. B. Keen, Cook & Co.

Electrolysis, in the treatment of stricture of the urethra. By Robert Newman, M.D.

*Scientific and Literary.*—Scribner's Monthly. This superb illustrated monthly excels even its past history in the beauty of its illustrations, and the choice character of its literature. It is among the purest and best. The tales of fiction found in its pages are always fresh and pure, being contributed by the best writers of the day. Every department is fully sustained. The editor, J. G. Holland, M.D., with his vigor and genius, embellishes its pages with gems of romance, besides enriching the editorial department with essays on numerous interesting subjects. Scribner & Co., New York. Price $4 00.

St. Nicholas—the juvenile magazine of the world—is the brightest, sunniest gem ever offered to the childish heart. Beaufully illustrated and loaded down with entertaining matter, drawn from the rich fields of fact and fiction, it is destined to warm and gladden the hearts of children everywhere. Every father should place it in the hands of his child, to instruct and amuse. Sanbury & Co , New York. $3 00 per annum.

Scientific American. This ably conducted journal enters upon a new year of prosperity. Its pages are well filled with the latest scientific news of the day, and copiously illustrated with recent investigations and improvements in art. It will be a welcome visitor in study and family circles. Mann & Co., 39 Park Row, New York. Price $3 00.

Scientific American Supplement. This supplement is a large weekly journal, devoted to science in all its departments. It will especially inform its readers of the various and useful improvements which will be exhibited at the Centennial. Its pages, with the most interesting and useful information, will afford the scientific student, and the reading man of the professions, a field of unusual value. It should be in the hands of all who desire to keep pace with the times. Mann & Co., 39 Park Row, New York. Price $5 00.

